# Association between circle of Willis and ischemic stroke: a systematic review and meta-analysis

**DOI:** 10.1186/s12868-021-00609-4

**Published:** 2021-01-21

**Authors:** Mohammed Oumer, Mekuriaw Alemayehu, Abebe Muche

**Affiliations:** 1 Department of Human Anatomy, School of Medicine, College of Medicine and Health Sciences, University of Gondar, Gondar, Amhara Ethiopia; 2 Department of Epidemiology, Institute of Public Health, College of Medicine and Health Sciences, University of Gondar, Gondar, Amhara Ethiopia; 3 Department of Environmental and Occupational Health and Safety, Institute of Public Health, College of Medicine and Health Sciences, University of Gondar, Gondar, Amhara Ethiopia

**Keywords:** Circle of willis, Ischemic stroke, Association, Systematic review and meta-analysis

## Abstract

**Background:**

Circle of Willis is the main structure that provides constant and regular blood flow to the brain, protects the brain from ischemia. Stroke has remained the second leading cause of death globally in the last fifteen years. It is the fifth leading cause of death in the United States. It is also the leading cause of serious adult disability. Interlinked problems related to ischemic stroke are become increasing nowadays. Strong evidence is needed about the pooled measure of association between the circle of Willis (COW) and ischemic stroke. Therefore, this systematic review and meta-analysis were intended to provide compressive and up to date evidence on the association between the variations of COW and ischemic stroke using the available studies.

**Methods:**

PubMed, Google Scholar, Science Direct, and Cochrane Library databases were systematically searched. All essential data were extracted using a standardized data extraction template. The heterogeneity across studies was assessed by using the Cochrane Q test statistic, I^2^ test statistic, and P-values. A fixed-effect model was used to estimate the pooled effect of the measure association between COW and ischemic stroke.

**Results:**

In this meta-analysis, 2,718 participants were involved. The pooled measure of association between COW and ischemic stroke was 1.38 (95% CI 0.87, 2.19). Therefore, this indicated that the presence of any variation in COW was 1.38 times more likely to develop ischemic stroke as compared to the patent COW. The presence of hypoplasia/incompleteness in a posterior communicating artery (PcomA) [Pooled OR: 1.34 (95% CI 0.80, 2.25)] and anterior communicating artery (AcomA) [Pooled OR: 1.32 (95% CI 0.81, 2.19)] were a contributing factor for the development of ischemic stroke. Hypertension was the most common comorbid condition, followed by diabetes mellitus, smoking, coronary artery disease, and hyperlipidemia.

**Conclusions:**

There was a non-significant positive association between COW variation and ischemic stroke in this meta-analysis.

## Background

Circle of Willis (COW) is defined as a vascular network formed at the base of the skull in the inter-peduncular fossa. Anatomically, its anterior part is constructed by the anterior cerebral artery (ACA), from either side. Additionally, the AcomA unites the right and left ACA. In the dorsal part of the COW, the unpaired basilar artery divides into the right and left posterior cerebral arteries (PCAs) and each connects to the bilateral internal carotid artery (ICA) through PcomAs [1–3]. The ACAs with middle cerebral arteries supply more than eighty percent of the cerebrum, while the rest part of it is provided by the PCAs [4]. Usually, the anatomical variations in the COW are observed [3, 5]. The COW, its function is to protect the brain from ischemia and infarction, is the main structure that provides constant and regular arterial blood flow into the brain [2]. Indeed, it is more sensitive to the lack of arterial blood and oxygen supply [5]. Even though the brain accounts for two percent of the body weight, it needs one-sixth of the cardiac output and one-fourth of oxygen in every breath. The continuous and constant supply of blood and oxygen is crucial for brain function [4, 5]. The pairs of two arteries (two vertebral and two ICAs with cerebral branches) provide the blood/oxygen flow into the areas of the brain. In the anterior surface of the brain, these arteries, with cerebral branches, form a cerebral arterial channel called the COW [3, 5]. Thomas Willis (from 1621–1675), the British anatomist and physician, was the first to observe the clinical importance of the COW and showed its physiologic function [6].

Globally, of the 56.9 million deaths in 2016, fifty-four percent were due to the top ten causes of mortality. Of them, the first and the second killers are ischemic heart attack and stroke, respectively. They accounted for a combined 15.2 million deaths. These diseases have stayed the leading cause of mortality in the previous fifteen years, globally [7].

Stroke is, in the United States of America, the fifth cause of mortality. Besides, it is the main cause of adult disability [8]. In this country, more than 795,000 persons face a stroke annually. Of these, 610,000 are found to be new stroke cases. Accordingly, every forty seconds a new case of stroke is detected and dies of a stroke per four minutes [8, 9]. Ischemic stroke responsible for more than eighty-seven percent of stroke cases and it can be subdivided into cardiogenic, atherosclerotic, lacunar, or cryptogenic sources [8, 10, 11]. The personal, social, and economic costs of ischemic stroke concerning the COW are substantial. Previously, several studies have revealed variations in the structure of the COW [5, 12, 13], and the sprouting of new vessels because of genetic and hemodynamic factors, the persistence of the arteries that normally disappear, and/or the disappearance that normally persist can be the variations [5, 12]. Importantly, the activity of the brain might not be influenced most of the time because of the presence of the collateral circulations, providing alternate routes for blood [5, 14, 15]. Besides, in cerebrovascular disease patients, COW can maintain adequate blood flow and reduce the damage of the affected areas through its potential redistribution role [16] and, notably, this compensation lies on the anatomical morphology of COW [16]. On the other way, the variations in the COW may alert cerebral hemodynamics and result in various cerebrovascular diseases. In particular, the formation of the cerebral aneurysm correlates with the morphology of COW [16, 17]. Ischemic stroke can also develop when there is a diminished cerebral blood flow due to severe stenosis of the vessels, the occlusion of the cerebral artery by embolism, or both occur simultaneously [18–21].

Interestingly, due to the emerging of medical imaging like Magnetic Resonance Angiography (MRA), clinical researches widely used to study the morphology and variation of COW [22]. The study done in China on a healthy population using these imaging techniques confirms the distribution of the variant types of COW, which provided the anatomical basis for future prognosis and treatment of cerebrovascular disease [16].

The variations of COW are clinically important because the COW has an essential role in cerebral hemodynamic as a collateral anastomotic network and persons with effective collateral circulations have a less risk of developing ischemic stroke as compared to those with ineffective collateral circulations [23–30].

The anatomical variations of COW have been proven to correlate with the formation of certain cerebrovascular diseases [1, 31, 32]. Although there are studies that study the variations of the anatomy of COW, it is not clear whether the presence of variation in the anatomy of COW is associated with ischemic stroke in a similar way among studies of different regions of the globe. Nowadays, interlinked problems related to cerebrovascular disease like ischemic stroke are become increasing. Strong evidence is needed about the pooled measure of association between COW and ischemic stroke. The finding of the review gives important evidence to the Health Care Professionals (Anatomists, Neurosurgeons, Medical students, for instance), Policy makers, Researchers, etc., and motivate them to give concern for it, to perform additional research, and to work with the risk factors. Thus, this systematic review and meta-analysis aimed to determine the pooled measure association between the anatomical variations of the COW and ischemic stroke using the available studies.

## Methods

The reporting of the current systematic review and meta-analysis followed the preferred reporting items for systematic reviews and meta-analysis statements (Additional file 1) [33].

### Study outcomes

In this review, assessing the association between COW and ischemic stroke was the primary outcome. Vessels in COW were defined as a patent, occlude, anomaly, incompleteness, or variation by using an imaging technique.

### Searching strategies

PubMed/Medline, PubMed Central, Science Direct, Google Scholar, and Cochrane Library databases were searched systematically for relevant studies. Sources including the websites of the American Stroke Association and American Heart Organization were retrieved. Besides, reference lists of identified studies were navigated for the presence of additional studies. The primary search was conducted in the PubMed database. The search in different databases was conducted using the following core search terms: “association”, “circle of Willis”, and “ischemic stroke”. Notably, Boolean operators (OR and AND) were applied for searching the mentioned key terms. The search in PubMed was conducted using the following search strategies: (“Association”) AND (“variation in circle of Willis” OR “anomaly in circle of Willis” OR “hypoplasia in circle of Willis” OR “stenosis in circle of Willis”) AND (“ischemic stroke”).

### Study eligibility criteria

The inclusion criteria for this review were studies published in the English language and carried out in any country. Published or unpublished full studies that reported a measure of association between the variation (anomaly, hypoplasia, or absent vessels) in COW and ischemic stroke were included.

When the information needed to consider eligibility was missing, animal-based experimental studies, cadaveric studies, and the study that did not report a direct association was excluded. Besides, the study participant’s diagnosis was not determined by imaging technique was excluded: CT imaging, CT angiography, magnetic resonance imaging, magnetic resonance angiography, and Trans-cranial color-coded Doppler Sonography.

### Assessment of methodological quality

In this review, the study quality was evaluated using the Newcastle–Ottawa Quality Assessment Scale for case–control and cohort studies (Additional file 2) [34]. This quality assessment scale has three sections: the first (focuses on selection), the second (comparability), and the third section (exposures, the outcome for cohort study). Using this checklist/scale, the two reviewers (MO and AM) independently assessed/appraised the quality of each study. Disagreements between reviewers were solved by taking the average score of the two reviewers. In the end, we considered good quality if the study scored six and above points on all quality assessment items.

### Data extraction and study selection

All essential data were extracted using a standardized data abstraction template. After the removal of duplicate articles, all eligible studies were screened based on title and abstract for possible inclusion. Full-text articles were screened and reviewed for the entirety to identify the final inclusion. Qualitative and quantitative data were extracted by two reviewers (MO and AM) from selected studies using a predetermined data collection template. Interestingly, publication year, main author, sample size, response rate, age of participants, study country, study design, duration of the study, sex of participants, the site of COW, imaging technique, branches of COW, the measure of association (odds ratio with its confidence interval), and other risk factors of ischemic stroke were included in the data abstraction template.

### Statistical analyses

In the present review, the data analyses were performed using STATA Version 14.1 Statistical Software. The data were extracted in Microsoft Excel (logarithm of the odds ratio and standard error of the logarithm of the odds ratio for each study was calculated) and exported into STATA for further analysis. The heterogeneity across studies was assessed by using the Cochrane Q test statistic (chi-square statistic with k-1 degree of freedom, P-values), and I^2^ (I-Squared) test statistic. It was considered as low, moderate, or high when the I^2^ test statistic result was 25%, 50%, and 75%, respectively [35]. This meta-analysis displayed that there is no statistically significant heterogeneity among studies (I^2^ = 0.0%, P-value = 0.972). Therefore, the fixed-effect model was applied to estimate the pooled effect of the measure of association between COW and ischemic stroke [36]. Visually, we used the Galbraith plot and Forest plot to assess the presence of heterogeneity across studies. Meta-cumulative of the measure of association between COW and ischemic stroke was presented. In all cases, a P-value of less than or equal to 0.05 was considered to be statistically significant. The findings of the meta-analysis were presented using the Forest plot and odds ratio with its 95% CI.

### Assessment of publication bias across studies

The publication bias was assessed by applying Egger’s regression test [37]. Significant publication bias was considered if a P-value became less than or equal to 0.05. Egger’s plot was presented to visualize the publication bias.

## Results

### Study selection

**Fig. 1:**
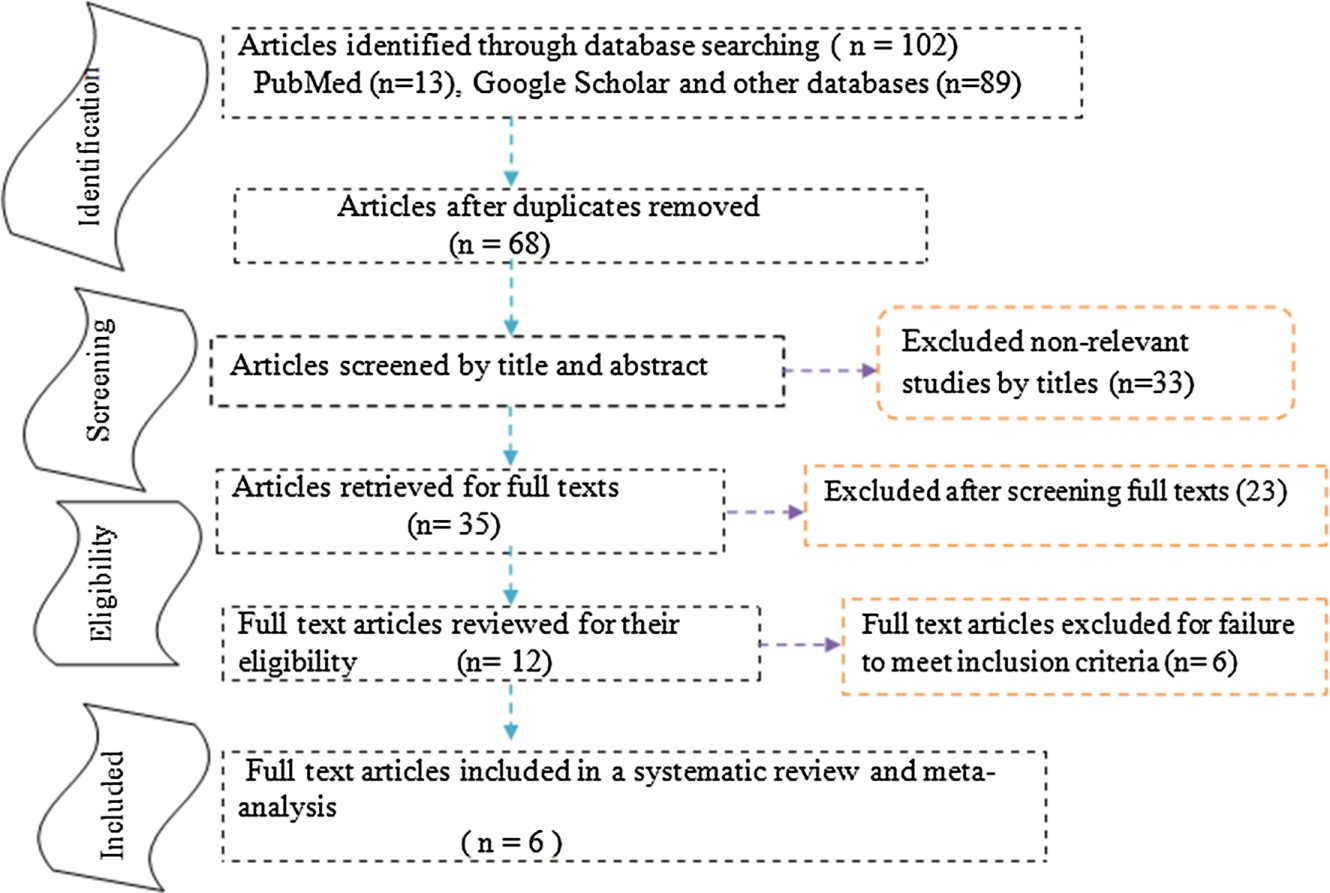
Study selection flow diagram; a figure. adapted from the Preferred Reporting Items for Systematic Reviews and Meta-Analyses group statement

A total of 102 articles were initially retrieved on the association between COW and ischemic stroke, of which thirty-four were excluded due to duplicated articles. Of sixty-eight articles, thirty-three articles were excluded after reviewing their titles and abstracts, it was non-essential. After screening and reviewing full-text articles, six full-text articles (n = 2718) were fulfilled the eligibility criteria and included in the systematic review and meta-analysis (Fig. 1).

### Characteristics of the original studies

All included studies were prospective cohort and case–control study design, two prospective cohort, and four case–control studies [17, 38–42]. The lowest and highest mean age of the respondents was 36 years and 68.9 years, respectively. The studies included participants, ranging from 120 to 976 [41, 42]. The largest study was carried out in Netherland. From all studies, two conducted in Netherland [40, 41], one in Taiwan [38], one in the United States [39], one in Poland [17], and one in Albania [42]. The total number of participants included in this study were 2718 participants (Table 1).

**Table 1:** Descriptive summary of the studies included in the meta-analysis for the association between the circle of Willis and ischemic stroke, 2020

S.no	Author	Publication year	Country	Sample size	Male sex	Mean age in years	Duration/ Follow-up time	Study design
1	Yu-Ming et al. [38]	2008	Taiwan	310	210	68.9	2006/l year	Case control
2	Shahan et al. [39]	2017	USA	457	–	36.0	2005–2015/10 years	Case control
3	Tom-Van et al. [40]	2016	Netherland	484	268	52.0	2009–2013/ 4 years	Prospective
4	SMART Study Group [41]	2015	Netherland	976	782	58.2	2001–2005/ 4 years	Prospective
5	Rafat et al. [17]	2015	Poland	371	265	66.0	2011–2015/ 4 yeas	Case control
6	Edlira et al. [42]	2014	Albania	120	52	60.2	2012–2013/ 1 year	Case control

### Quality of the studies

By using the Newcastle–Ottawa Quality Assessment Scale criteria, the quality score of the included studies was ranged between six-point and nine-points. All studies scored above six points. Therefore, no studies were included that had poor quality in this review.

### Characteristics of studies based on imaging technique and sites

Most studies were used MRI and MRA to diagnose the status of COW among the participants (Table 2).

**Table 2:** Imaging technique that studies used and the site determined in the circle of Willis, 2020

Authors	Imaging technique	Sites
Yu-Ming et al. [38]	MRI and MRA	Circle of Willis, PcomA
Shahan et al. [39]	CT, CTA, MRI, and MRA	Circle of Willis, ICA
Tom-Van et al. [40]	NCCT, CTA, and CTP	Circle of Willis, MCA, PcomA
SMART Study Group [41]	TOF-MRA and MRA	Circle of Willis, AcomA
Rafat et al. [17]	TCCD	Circle of Willis, AcomA
Edlira et al. [42]	CTA	Circle of Willis

*MRI* Magnetic Resonance Imaging, *TOF-MRA* Time of flight Magnetic resonance Angiography, CTA Computed Tomography Angiography, *CTP* Computed Tomography Perfusion, *NCCT *Non-Contrast CT, *TCCD* Transcranial color-coded ultra Sonography

### Predisposing risk factors of ischemic stroke

Hypertension was the most common comorbid condition across the studies, followed by diabetes mellitus, smoking, coronary artery disease, and hyperlipidemia. The mean age of ischemic stroke patients was significantly higher than the control groups [42] (Table 3).

**Table 3:** Predisposing risk factors for ischemic stroke patients across different studies, 2020

S.no	Author/ year of publication	Predisposing risk factors
1	Yu-Ming et al. [38]	HT, DM, AF, OC, smoking, and other cardiac diseases
2	Shahan, et al. [39]	Motor vehicle accident and treated with heparin
3	Tom-Van et al. [40]	HT, DM, AF, and smoking
4	SMART Study Group [41]	HT, DM, CAD, PAD,HPL, and smoking
5	Rafat et al. [17]	HT, DM, smoking, and ischemic heart disease
6	Edlira et al. [42]	Age and CAD

*HT* Hypertension, *DM* Diabetes Mellitus, *CAD* Coronary Artery Disease, *PAD* Peripheral Artery Disease, *HPL* Hyperlipidemia, *AF *Atrial Fibrillation, *OC* Oral Contraceptive

### The association between the circle of Willis and ischemic stroke

In this systematic review and meta-analysis, the odds ratio, 95% confidence interval (lower–upper bound), P-values, and their summary results concerning the association between COW and ischemic stroke are described (Table 4).

**Table 4:** Studies reported the association between the circle of Willis and ischemic stroke, 2020

S.no	Author	Odds ratio	95% CI	P-value	Comments
1	Yu-Ming et al	3.21	1.43–9.62	0.036	PcomA hypoplasia in ischemic stroke patients was 19.35%, which was significantly higher than the control group (8.20%)
2	Shahan et al	7.1	1.28–33.3	0.001	The presence of an additional artery will reduce the risk of stroke
3	Tom-Van et al	1.9	1.3–3.0	0.02	Stenosis of proximal MCA or an incomplete posterior COW was determinants of poor collateral flow in acute stroke patients
4	SMART Group	2.8	1.3–6.3	0.01	An incomplete anterior COW was a predictor of anterior circulation stroke
5	Rafat et al	1.28	1.16–1.49	0.001	The presence of AcomA and PcomA reduced the risk of stroke by 72%
6	Edlira et al	1.5	0.6–3.8	0.4	Anatomical variations of COW in stroke patients were 23.3%, which was higher than the control group (16.7%)

### Meta-analysis

**Fig. 2:**
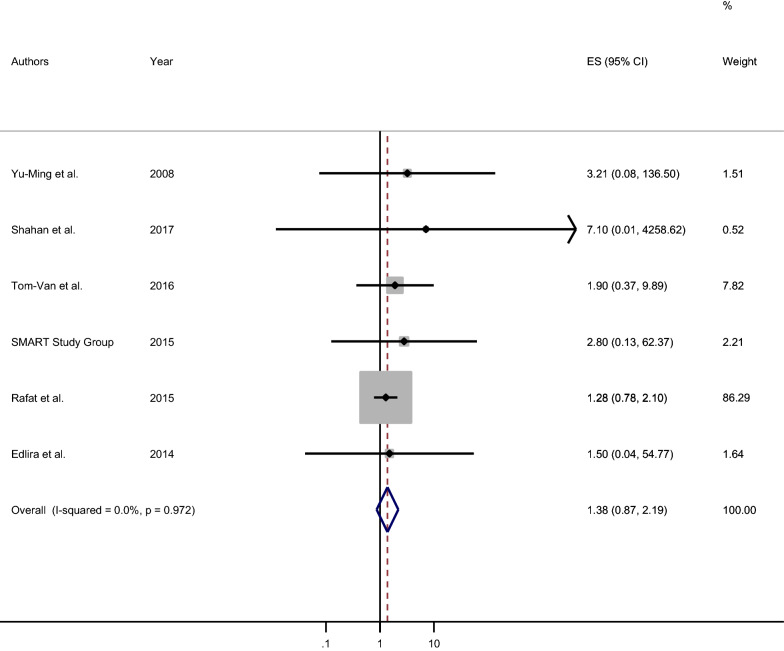
Forest plot of the pooled measure of association between the circle Willis and ischemic stroke, 2020

In this meta-analysis, six original studies, 2718 participants were involved to estimate the pooled effect measure between COW and ischemic stroke. Therefore, the pooled odds ratio was 1.4 (Fig. 2). Statistically significant heterogeneity was not detected across the studies (I^2^ = 0.0%, P-value = 0.972) [43]. As a result, the fixed-effect model was used to estimate the pooled effect of the measure of association between COW and ischemic stroke. There was a non-significant positive association between variation in COW and ischemic stroke [Pooled OR: 1.38 (95% CI 0.87, 2.19)]. The participants with variation, incompleteness, or hypoplasia in any part of the circle of Willis were 1.4 times more likely to develop ischemic stroke as compared to their counterparts (Fig. 2).

**Fig. 3:**
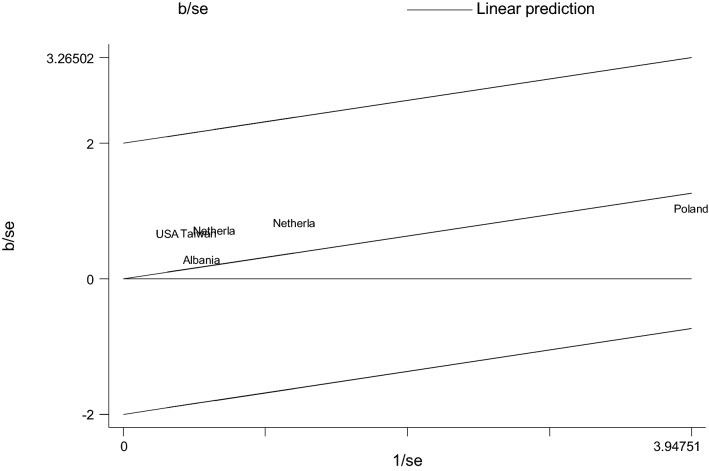
Galbraith plot showing the variability of individual measure of association between the circle Willis and ischemic stroke by study country, 2020

The Galbraith plot also visualized all studies based on their country and it indicated that there is no variability between the studies as studies are located within its confidence interval limits (Fig. 3). The Galbraith plot provided a graphical display of the amount of heterogeneity from a meta-analysis. This plot displayed the coefficient over the standard error of the coefficient is plotted against the inverse of the standard error, known as the study precision. The position of each study country on the horizontal axis indicated the weight allocated to it in a meta-analysis while the position on the vertical axis gave the contribution of each study to the Q statistic for heterogeneity. To say homogeneous, all studies or countries should lie within the 95% confidence bounds, positioned two units over and below the regression line (or lie between − 2 and 2 units) (Fig. 3).

### Meta-cumulative analysis

**Fig. 4:**
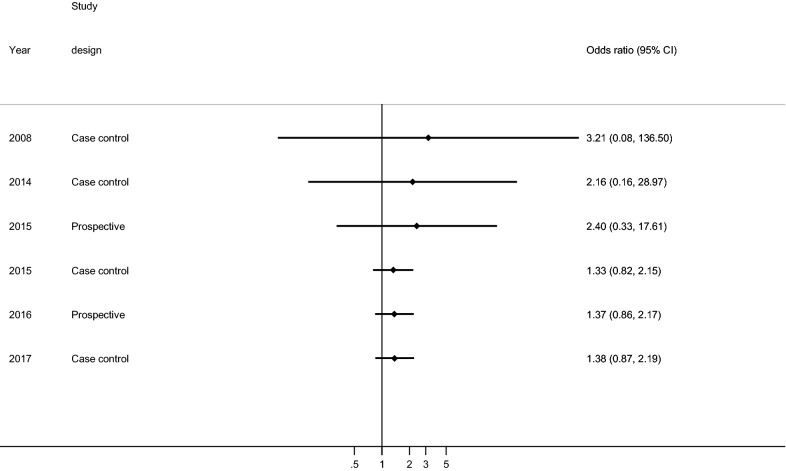
Meta-cumulative of the measure of association between the circle Willis and ischemic stroke by study country, 2020

This study describes the cumulative effect of the association between COW and ischemic stroke from 2008 to 2017. The significance of the association was increased successively from each year (Fig. 4).

### Association between variation in the posterior communicating artery and ischemic stroke

**Fig. 5:**
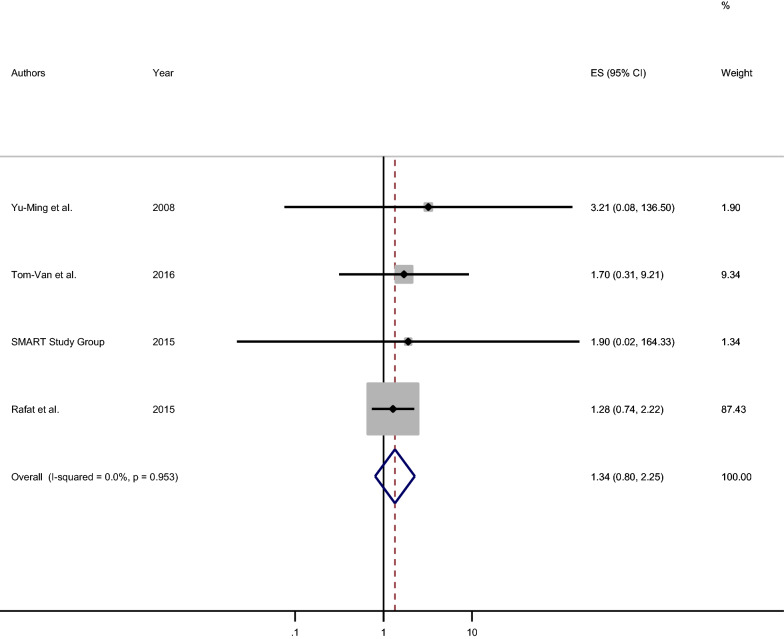
Forest plot of the pooled measure of association between hypoplasia of posterior communicating artery and ischemic stroke, 2020

The association between hypoplasia (or incomplete PcomA) of PcomA and ischemic stroke was also estimated using a fixed-effect model among four studies (I^2^ = 0.0%, P-value = 0.953). The statistically non-significant association between variation in PcomA and ischemic stroke was observed [pooled OR: 1.34 (95% CI 0.80, 2.25)]. The respondents who had hypoplasia or incomplete PcomA were 1.3 times more likely to develop ischemic stroke as compared to those who free from hypoplasia/incompleteness (Fig. 5).

### Association between variation in the anterior communicating artery and ischemic stroke

**Fig. 6:**
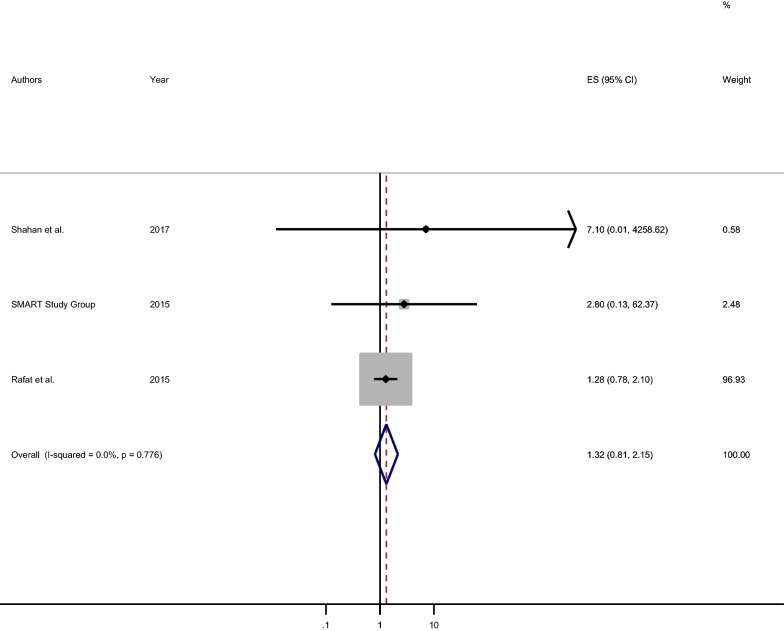
Forest plot of the pooled measure of association between variation in anterior communicating artery and ischemic stroke, 2020

The association between variation (like incompleteness) in AcomA and ischemic stroke was estimated and the statistically non-significant positive association was detected [pooled OR: 1.32 (95% CI 0.81, 2.19)]. The participants who had variation (incompleteness) in AcomA were 1.3 times more likely to develop ischemic stroke as compared to those who had a patent AcomA (Fig. 6).

**Fig. 7:**
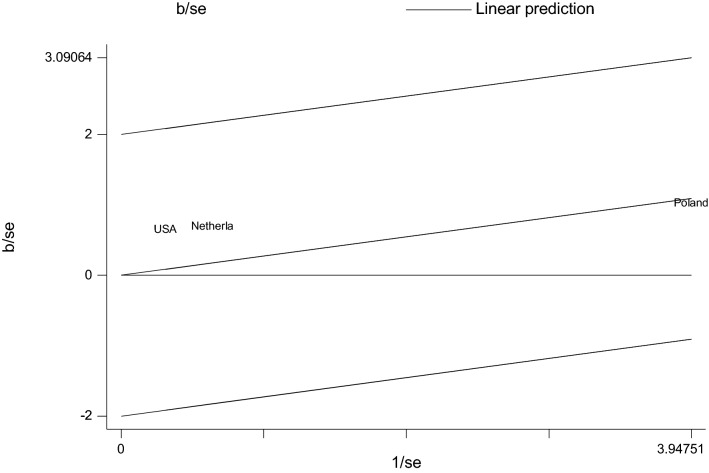
Galbraith plot that shows the variability in measure of association between variation in anterior communicating artery and ischemic stroke, 2020

The Galbraith plot visual display showed that the non-variability between studies (Fig. 7).

## Discussion

This systematic review and meta-analysis were employed to determine the association between the variation of COW and ischemic stroke. Stroke is one of the leading causes of death and disability in the United States. The American Heart Association and American Stroke Association estimated the number of new strokes that occurred each year has become increasing [10]. It may not be different in this regard in developing countries too. Even though there are some studies done in some countries that provide data on the association between COW and ischemic stroke, very few of these gave a direct measure association with dichotomous outcomes including cases and controls or exposed and unexposed, which allow comparability between the studies. The data in this review provide estimates of the variational impact of COW on ischemic stroke in different countries of the world.

In this systematic review and meta-analysis, a total 2718 participants were included to estimate the pooled measure of association. In most studies, hypertension was the most common comorbid condition in groups, followed by diabetes mellitus, smoking, hyperlipidemia, and coronary artery disease [17, 38, 40–42, 44].

This review showed there was no heterogeneity across the studies. A fixed-effect model estimated that the participants with variation, incompleteness, or hypoplasia in any part of the circle of Willis were 1.4 times more likely to develop ischemic stroke as compared to those participants who had the normal anatomy of the circle of Willis. This finding is in agreement with the reviews conducted by the American Heart Association [45, 46], having internal carotid artery stenosis may predispose to the development of ischemic stroke.

The time trend of the significance and the association increases over time due to the accumulative effect of studies over the successive year. The pooled effect of the measure of association was relatively free from the publication bias.

In this review, we tried to associate COW variants and ischemic stroke. The main anatomical variants of COW are hypoplasia (for instance, hypoplasia of the PcomA, the AcomA, the circular part of the PCA, or the circular part of the ACA), absent vessels (absent vessels of one or other PcomAs), anomalous origin (persistence of the embryonic derivation of the PCA from the ICA), or accessory vessels (duplications or triplications of one of the components of the COW) [42]. The PcomA is a principal collateral circulation pathway and the source of numerous penetrating arteries that supply the ventrolateral and dorsomedial thalamic nuclei, as well as the lateral aspect of the thalamic pole, cerebral peduncle, tuber cinereum, and mammillary bodies. PcomA hypoplasia is a congenital variant of the COW characterized by a narrow, underdeveloped PcomA with restricted blood flow [38]. As reviewed articles have shown, the PcomA hypoplasia is associated with the risk of ischemic stroke, even in the absence of ICA occlusion. The most common ischemic event, in those who had PcomA hypoplasia, was ipsilateral thalamic lacunar infarctions with or without occipital lobe involvement [38]. However, in the case of fetal type PcomA (when the PcomA has a larger diameter than the first segment of the PCA), Shahan et al. reported that an enlarged PcomA (persistent fetal-type circulation) may improve collateralization between the anterior (carotid) and posterior (vertebral) circulations, this may decrease the risk of stroke [39]. On the other hand, an incomplete anterior COW combined with an incomplete posterior COW is associated with anterior circulation stroke. An incomplete anterior COW and a one-sided or two-sided incomplete posterior COW are positively associated with anterior circulation stroke [41].

The variations of COW are clinically important. It plays an important role in cerebral hemodynamics and collateral anastomotic network. Therefore, individuals who have effective collateral circulations are less at risk of developing ischemic stroke than those with ineffective collateral circulations [23–26, 28, 29].

### Strength and limitations of the review

This review provided cumulative evidence on qualitative and quantitative data between the variation in the circle of Willis and ischemic stroke; give a better understanding between the nature of the circle of Wills and the development of the disease.

The review limitation was the consideration of studies published in English. Besides, this meta-analysis represented only the studies from the six countries.

## Conclusions

This systematic review and meta-analysis showed that there was a non-significant positive association between the variation of the circle of Willis and ischemic stroke.

Therefore, the authors recommend that, special awareness creation for the people to focus on primary prevention by undergoing early screening about the status of circle Willis through imaging technique.

By doing so, the stroke-prone individuals will be identified and targeted for particular interventions. Prevention remains the cornerstone of therapy for these devastating diseases.

## Supplementary Information

**Additional file 1.** PRISMA checklist.

**Additional file 2.** Newcastle–Ottawa Quality Assessment Scale.

## Data Availability

The data sets used and/or analyzed during the current systematic review and meta-analysis are presented within the manuscript and its Additional files 1, 2.

## Abbreviations

AcomA: Anterior communicating artery
ACA: Anterior cerebral arteries
COW: Circle of willis
ICA: Internal carotid artery
MRA: Magnetic resonance angiography
MCA: Middle cerebral arteries
PCA: Posterior cerebral arteries
PcomA: Posterior communicating arteries
