# Supplementary material for: Association between circle of Willis and ischemic stroke: a systematic review and meta-analysis
Source: BMC Neurosci. 2021 Jan 21;22:3. doi: 10.1186/s12868-021-00609-4 (PMC7818725; doi:10.1186/s12868-021-00609-4)
Supplement: Supplementary file 2 — Additional file 2. Newcastle–Ottawa Quality Assessment Scale. [file 12868_2021_609_MOESM2_ESM.docx]

1. **Newcastle-Ottawa Quality Assessment Scale (Adapted for Cross-sectional studies)**

**Selection: (Maximum 3 stars)**

1. Representativeness of the sample:

a) Truly representative of the average in the target population. ⃰ (all subjects or random sampling)

b) Somewhat representative of the average in the target population. ⃰ (non-random sampling)

c) Selected group of users.

d) No description of the sampling strategy.

2. Non-respondents:

a) Comparability between respondents and non-respondents characteristics is established, and the response rate is satisfactory. ⃰

b) The response rate is unsatisfactory, or the comparability between respondents and non-respondents is unsatisfactory.

c) No description of the response rate or the characteristics of the responders and the non- responders.

3. Ascertainment of the exposure (risk factor):

a) Validated measurement tool. ⃰

b) Non-validated measurement tool, but the tool is available or described.

c) No description of the measurement tool.

**Comparability: (Maximum 2 stars)**

1. The subjects in different outcome groups are comparable, based on the study design or analysis.

Confounding factors are controlled.

a) The study controls for the most important factor (select one). ⃰

b) The study control for any additional factor. ⃰

**Outcome: (Maximum 2 stars)**

1**.** Assessment of the outcome:

a) Independent blind assessment. ⃰

b) Record linkage. ⃰

c) Self report.

d) No description.

2. Statistical test:

a) The statistical test used to analyze the data is clearly described and appropriate, and the

measurement of the association is presented, including confidence intervals and the probability level (p value). ⃰

b) The statistical test is not appropriate, not described or incomplete

1. **Newcastle-Ottawa Quality Assessment scale** **(for case control studies)**

Note: A study can be awarded a maximum of one star for each numbered item within the Selection and Exposure categories. A maximum of two stars can be given for Comparability.

**Selection**

1. Is the case definition adequate?

a) Yes, with independent validation ⃰

b) Yes, e.g. record linkage or based on self-reports

c) No description

2. Representativeness of the cases

a) Consecutive or obviously representative series of cases ⃰

b) Potential for selection biases or not stated

3. Selection of controls

a) Community controls ⃰

b) Hospital controls

c) No description

4. Definition of Controls

a) No history of disease (endpoint) ⃰

b) No description of source

**Comparability**

1. Comparability of cases and controls on the basis of the design or analysis

a) Study controls for _______________ (Select the most important factor.) ⃰

b) Study controls for any additional factor ⃰ (This criteria could be modified to indicate specific control for a second important factor.)

**Exposure**

1. Ascertainment of exposure

a) Secure record (e.g. surgical records) ⃰

b) Structured interview where blind to case/control status ⃰

c) Interview not blinded to case/control status

d) Written self-report or medical record only

e) No description

2. Same method of ascertainment for cases and controls

a) Yes ⃰

b) No

3. Non-Response rate

a) Same rate for both groups ⃰

b) Non respondents described

c) Rate different and no designation

1. **Newcastle-Ottawa Quality Assessment Scale (for cohort studies)**

Note: A study can be awarded a maximum of one star for each numbered item within the Selection and Outcome categories. A maximum of two stars can be given for Comparability

**Selection**

1. Representativeness of the exposed cohort

a) Truly representative of the average _______________ (describe) in the community ⃰

b) Somewhat representative of the average ______________ in the community ⃰

c) Selected group of users eg nurses, volunteers

d) No description of the derivation of the cohort

2. Selection of the non-exposed cohort

a) Drawn from the same community as the exposed cohort ⃰

b) Drawn from a different source

c) No description of the derivation of the non-exposed cohort

3. Ascertainment of exposure

a) Secure record (e.g. surgical records) ⃰

b) Structured interview ⃰

c) Written self-report

d) No description

4. Demonstration that outcome of interest was not present at start of study

a) Yes ⃰

b) No

**Comparability**

1. Comparability of cohorts on the basis of the design or analysis

a) Study controls for _____________ (select the most important factor) ⃰

b) Study controls for any additional factor ⃰ (This criteria could be modified to indicate specific control for a second important factor.)

**Outcome**

1. Assessment of outcome

a) Independent blind assessment ⃰

b) Record linkage ⃰

c) Self-report

d) No description

2. Was follow-up long enough for outcomes to occur

a) Yes (select an adequate follow up period for outcome of interest) ⃰

b) No

3. Adequacy of follow up of cohorts

a) Complete follow up - all subjects accounted for ⃰

b) Subjects lost to follow up unlikely to introduce bias - small number lost - > ____ % (select an adequate %) follow up, or description provided of those lost) ⃰

c) Follow up rate < ____% (select an adequate %) and no description of those lost

d) No statement

**Quality** **score:**

Good quality: ≥ 6 points

Poor quality: < 6 points
